# Correlation between serum inflammatory factors and psychosomatic syndrome in patients with chronic obstructive pulmonary disease: an observational study

**DOI:** 10.3389/fpsyt.2025.1586399

**Published:** 2025-07-10

**Authors:** Wenjing Zhang, Yujuan Cui, Ling Sun, Chunlin Tu, Yanfang Yu

**Affiliations:** Department of Respiratory and Critical Care Medicine, Jiading District Central Hospital Affiliated Shanghai University of Medicine & Health Sciences, Shanghai, China

**Keywords:** COPD, IL-6, MMRC, cat, DCPR

## Abstract

**Background:**

Chronic obstructive pulmonary disease (COPD) is increasingly prevalent in respiratory medicine, with rising incidence and mortality rates annually. Beyond respiratory implications, it leads to cardiovascular events, osteoporosis, muscle loss, and psychosomatic syndrome, often overlooked yet pivotal in COPD prognosis. Despite this, the relationship between COPD and psychosomatic syndrome remains unclear. This study aimed to explore the correlation between acute exacerbation of chronic obstructive pulmonary disease (AECOPD) in patients and psychosomatic syndrome, alongside peripheral blood inflammatory factors and symptom burden (e.g., modified Medical Research Council [mMRC] and COPD assessment test [CAT]). Identifying high-risk AECOPD patients with psychosomatic syndrome through these markers could enable early intervention and improve prognosis.

**Methods:**

This observational study recruited 202 AECOPD patients admitted to the Respiratory and Critical Care Medicine Department of Shanghai Jiading Central Hospital from March 1st, 2022 and May 1st, 2024. After obtaining consent, we collected demographic data, blood routine results, albumin, prealbumin, lung function metrics, mMRC, CAT scores, and other parameters. We evaluated psychiatric comorbidities using the revised version of the Diagnostic Criteria for Psychosomatic Research (DCPR-R). Binary logistic regression and ROC curve analyses were employed to assess the correlation between inflammatory factors, symptom burden, and psychosomatic syndrome, as well as their diagnostic and predictive value for psychosomatic syndrome.

**Results:**

Among 202 AECOPD patients, 144 were in the DCPR (+) group and 58 in the DCPR (-) group. The DCPR (+) group had higher white blood cell counts, neutrophil counts, monocyte counts, interleukin (IL)-6 levels, mMRC scores, and CAT scores than those of the DCPR (-) group, (P < 0.05), Albumin and prealbumin levels were significantly lower in the DCPR (+) group than those in the DCPR (-) group (P < 0.05). There were no significant differences between the two groups in terms of age, sex, marital status, years of education, body mass index, smoking history, drinking history, diabetes, hypertension, PaCO_2_, PaO_2_, neutral ratio, lymphocyte count, monocyte ratio, FEV_1_% pred, and FEV_1_/FVC% (P > 0.05). Multivariate binary logistic regression analysis identified IL-6, mMRC, and CAT score as independent risk factors for psychosomatic syndrome in AECOPD patients. The odds ratios (OR) and 95% confidence intervals (CI) were: IL-6: OR=1.192 (95% CI: 1.091-1.302), P < 0.001; mMRC: OR=1.922 (95% CI: 1.175-3.144), P = 0.009; CAT: OR=1.149 (95% CI: 1.073-1.231), P < 0.001.

**Conclusions:**

Peripheral blood IL-6, mMRC, and CAT were independent risk factors for psychiatric comorbidities in AECOPD patients, with good predictive value for psychosomatic integration. Subjective symptoms including cough, phlegm, sleep disturbances, lack of confidence in going out, and difficulty breathing contribute more to psychiatric comorbidities than the objective indicator of FEV_1_.

## 1 Background

Chronic obstructive pulmonary disease (COPD) is an inflammatory disease of the airways characterized by progressive airflow limitation. Its main symptoms include cough, sputum production, and progressively worsening breathing difficulties (1). The primary risk factors include smoking, air pollution, and occupational exposure (2). As of 2017, COPD has become the third leading cause of death globally (3). COPD patients experience a prolonged course marked by recurrent exacerbations, slow progression, and gradual deterioration. This condition not only impacts the respiratory system but also heightens the risk of cardiovascular events, osteoporosis, muscle loss, anxiety, depression, cognitive decline, and other systemic and multisystem impairments (4–6). Additionally, the prevalence of psychological disorders is relatively high in COPD patients. According to the meta-analysis conducted by Matte et al. (7) and Zhang et al. (8), the prevalence of depression among COPD patients is reported as 24.6% and 27.1%, respectively, while in non-COPD patients, the prevalence of depression is 11.7% and 10%, respectively. Reportedly, the incidence of cognitive impairment in COPD patients is 17-56.7%, whereas in healthy controls, it is 12-16.7% (9). However, healthcare workers often lack sufficient understanding of COPD, leading to frequent misdiagnosis and improper management, which in turn result in poor health outcomes and prognosis. Rahi et al. pointed out that depression or anxiety consistently increases the risk of adverse outcomes in COPD patients (odds ratio [OR]=1.43; 95% confidence interval [CI]: 1.22-1.68). Specifically, depression increases the risk of death by 1.83 (95% CI: 1.00-3.36), and anxiety heightens the risk of death by 1.27 (95% CI: 1.02-1.58) (10). Anxiety and depression are independent risk factors of death in COPD patients (11), and depression exacerbates their deterioration (12). An increase in hospitalization duration owing to acute exacerbation, decreased lung function, reduced 6-min walking distance, and decreased quality of life has been observed in COPD patients with anxiety or depression (13). Evidently, the relationship among COPD, anxiety, and depression is bidirectional (14). Anxiety and depression have adverse effects on the prognosis of COPD. COPD patients are at an increased risk of anxiety and depression owing to breathing difficulties, partial loss of social function, and economic burden (6). However, the reality is more intricate than commonly perceived.

In COPD, a chronic inflammatory disease, interleukin (IL)-6 and IL-1β (crucial airway inflammatory cytokines) play key roles in disease progression (15). He et al. confirmed that IL-6 accelerates the rate of FEV_1_ decline in COPD patients who smoke (16). Evidence from both animal and clinical studies suggests that elevated levels of the pro-inflammatory cytokine IL-6 in the peripheral blood or central nervous system play an important role in depression (17). Rizzo et al. induced depression-like behavior in mice through intraventricular injection of recombinant IL-6 (18). Long et al. confirmed a close correlation between serum IL-6 levels and depression in COPD patients (19). Zhang et al.’s study showed that the levels of IL-6, IL-8, IL-10, and tumor necrosis factor-α were significantly higher in the COPD group with depression than in the non-depression group (P < 0.05) (20).

COPD patients who experience both anxiety and depression are at increased risk of acute exacerbations, hospitalizations, and mortality (21, 22). Therefore, respiratory physicians aim to promptly identify COPD patients with psychiatric comorbidities, intervene timely, and improve prognosis. Traditional diagnostic criteria for psychiatric comorbidities are challenging to apply to these patients, often yielding low positivity rates, and many subthreshold mental health conditions are prone to misdiagnosis. The Diagnostic Criteria for Research in Psychology (DCPR) are considered supplementary tools for identifying subthresholds or unclassified psychiatry associated with physical diseases (23, 24). The revised version of the DCPR (DCPR-R) Semi-Structured Interview assesses 14 psychosomatic syndromes: health anxiety, disease phobia, hypochondriasis, thanatophobia, illness denial, persistent somatization, conversion symptoms, anniversary reaction, demoralization, irritable mood, somatic symptoms secondary to a psychiatric disorder, type A behavior, alexithymia, and allostatic overload (25–27).

However, the association between psychosomatic syndrome and pro-inflammatory factors such as IL-6, CRP, and PCT in the peripheral blood of acute exacerbation of chronic obstructive pulmonary disease (AECOPD) patients is still unclear. Considering the promoting role of IL-6 in depression among COPD patients, we hypothesize an intrinsic link between IL-6 and psychosomatic syndromes. Our aim was to identify high-risk COPD patients for psychosomatic syndromes as early through simple IL-6 detection, implement psychological interventions, improve patient prognosis, and alleviate disease burden.

## 2 Method

### 2.1 Study participants and consent to participate

**Figure 1:**
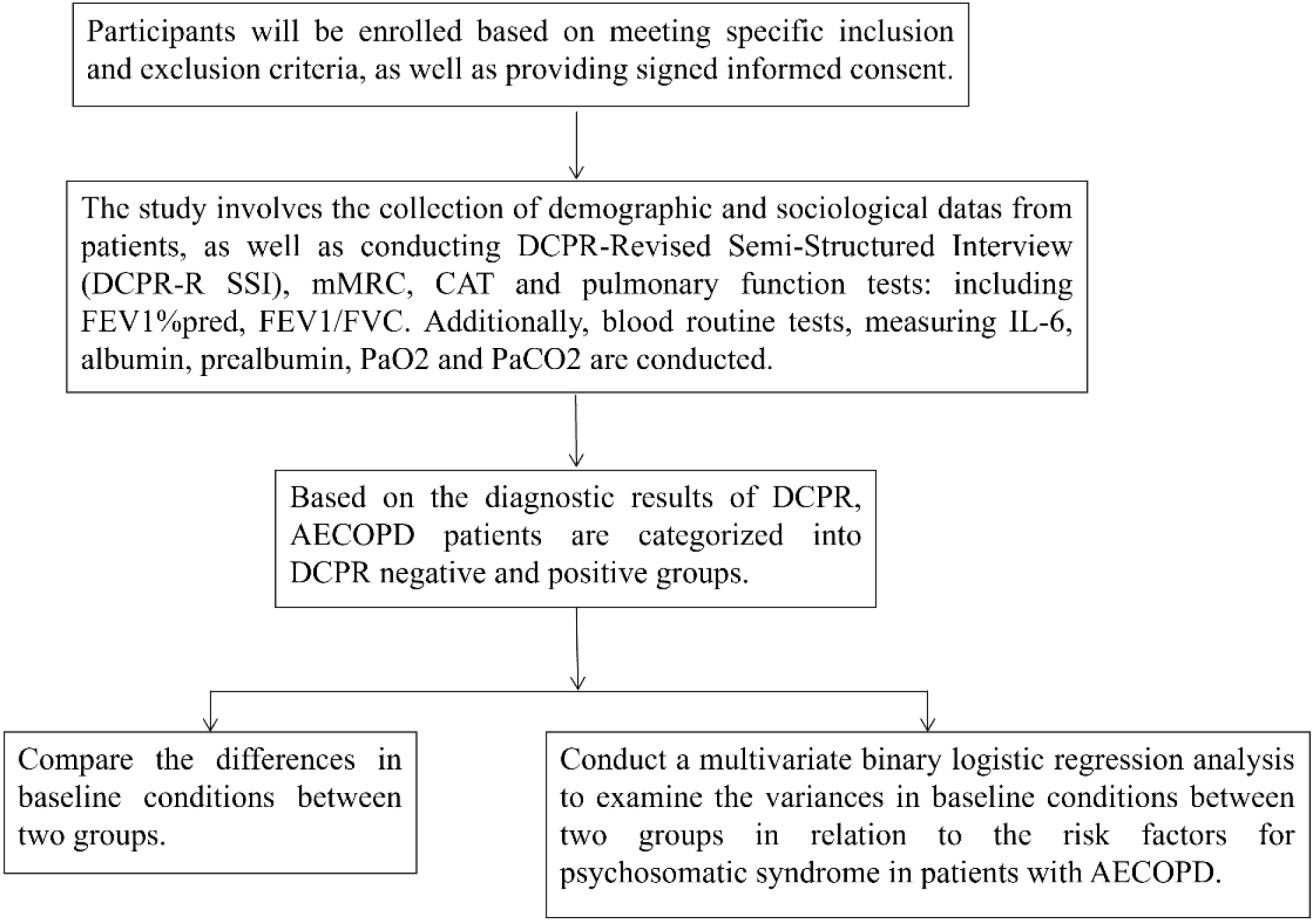
Research flowchart. DCPR, The Diagnostic Criteria for Psychosomatic Research; mMRC, modified Medical Research Council; CAT, COPD assessment test; AECOPD, Acute exacerbation of Chronic Obstructive Pulmonary Disease; IL-6, Interleukin 6; FEV_1_, Forced Expiratory Volume in 1 s; FEV_1_%pred, The percentage of predicted values of FEV_1_; PaO_2_, The partial pressure of arterial oxygen; PaCO_2_, The partial pressure of arterial carbon dioxide.

AECOPD patients admitted to the Respiratory and Critical Care Medicine Department of Shanghai Jiading Central Hospital (Jiading District Central Hospital Affiliated Shanghai University of Health and Medicine) between March 1st, 2022 and May 1st, 2024 were considered for inclusion in this study. All participants provided written informed consent (As shown in **Figure 1**).

### 2.2 Ethical support

This study has been approved by the Research Ethics Committee of Shanghai Jiading Central Hospital (2022K05).

### 2.2 Inclusion criteria

According to the 2023 Global Initiative for Chronic Obstructive Lung Disease guidelines (28), patients are diagnosed with COPD if they meet the following criteria: (1) age ≥ 40 years old; (2) post-bronchodilator (salbutamol) inhalation, lung function FEV_1_/FVC<70%; (3) ability to read and write Chinese, and communicate effectively with healthcare providers; (4) no systemic glucocorticoids use in the past 4 weeks; (5) willingness to provide voluntary and signed informed consent.

### 2.3 Exclusion criteria

(1) Patients receiving long-term oral corticosteroid treatment (≥ 4 weeks); (2) patients with severe liver and kidney dysfunction, hematological malignancies, or other malignant tumors; (3) patients with intellectual disability, confusion, or difficulty writing.

### 2.4 Flowchart

### 2.4 Process and tools used

#### 2.4.1 General data

Demographic and sociological data of the patients were collected, including age, sex, marital status, years of education, BMI, smoking history, alcohol consumption, presence of diabetes, and hypertension.

#### 2.4.2 Psychosomatic medicine interviews

Experienced physicians, who had undergone systematic training and were familiar with the diagnostic criteria for psychosomatic medicine, conducted interviews with AECOPD patients included in the study using a semi-structured diagnostic tool for psychosomatic medicine (DCPR-R-SSI), which corresponded to the DCPR-R. The DCPR-R-SSI focuses on the preceding 6–12 months and comprises 79 items answered with yes/no (29). It assesses 14 psychosomatic syndromes across four diagnostic modules (stress, illness behavior, psychological manifestations, and personality). The stress module covers allostatic overload, representing the cumulative effects of stressful experiences. Illness behavior encompasses hypochondriasis, disease phobia, thanatophobia, health anxiety, persistent somatization, conversion symptoms, anniversary reactions, and illness denial. Psychological manifestations include demoralization, irritable mood, and secondary somatic symptoms. The personality module addresses type A behavior and alexithymia (27). Research conducted domestically substantiates the suitability of DCPR for clinical implementation within China (30, 31).

#### 2.4.3 Determination of blood routine tests and levels of IL-6, albumin, prealbumin, PaO_2_, PaCO_2_

On the morning of admission, venous blood was collected from patients on an empty stomach using a disposable blood collection vessel was with EDTA as the anticoagulant. A total of 2 ml of blood was collected. Blood routine analysis was conducted using the French ABXPENTRA 120 DF automatic blood analyzer, which employs flow cytometry and impedance spectroscopy. Additionally, 3 ml of venous blood was collected on an empty stomach and centrifuged at 3000 rpm for 10 min. The serum was then collected for further analysis. A Chinese Hotgen fully automated chemiluminescence analyzer C3000 was used to detect IL-6 using a magnetic particle chemiluminescence immunoassay. The Abbott ARCHITECT C16000 fully automated biochemical analyzer was utilized to detect albumin employing the bromocresol green method, and prealbumin was detected using the immunoprojection turbidity method. On the day of admission, 1.5 ml of arterial blood was extracted from patients, and PaO_2_ and PaCO_2_ were detected using an ABL80 SC80 blood gas analyzer (Redumit, Denmark).

#### 2.4.4 Pulmonary function measurement

According to the guidelines of the American Thoracic Society and European Respiratory Society, lung function tests were conducted using the JaegerMaster Screen lung function testing system for vital capacity, whole-body plethysmography, and pulmonary diffusion function measurements. The recording personnel were professionally trained physicians. The forced expiratory volume (FEV_1_/FVC), one-second rate (FEV_1_/FVC), and percentage of forced expiratory volume in one second to the expected value (FEV_1_% pred) were recorded.

#### 2.4.5 Modified Medical Research Council

The mMRC scale is a self-rating tool used to measure the degree of disability caused by breathlessness in daily activities, rated on a scale from 0 to 4: 0, no breathlessness except with strenuous exercise; 1, shortness of breath when hurrying on the level or walking up a slight hill; 2, walks slower than people of same age on the level because of breathlessness or has to stop to catch breath when walking at their own pace on the level; 3, stops for breath after walking ∼100m or after few minutes on the level; and 4, too breathless to leave the house, or breathless when dressing or undressing (32).

#### 2.4.6 COPD assessment test

CAT is a questionnaire designed to measure the health status of COPD patients. Eight statements assess the best- and worst-case scenarios for coughing, phlegm, chest tightness, breathlessness when going up hills/stairs, activity limitations at home, confidence in leaving home, sleep, and energy. Each statement is scored from 0 to 5 (best to worst), with the total score ranging from 0 to 40 (33).

### 2.5 Statistical analysis

This study builds upon prior research and employs G*Power software to determine the appropriate sample size. An effect size of 0.3 was utilized, with a statistical power of 0.8 and a significance level of 0.05. The calculated minimum sample size required was 180 participants. To account for a potential dropout rate of 10%, a total of 202 patients were ultimately recruited.

The experimental data were established in a database using Windows Excel software, and statistical analysis and plotting were performed using SPSS 26.0 and GraphPad Prism software (version 8.0) for data processing. Measurement data that conform to a normal distribution are represented by mean ± standard deviation (X ± S), while non-normal distribution measurement data are expressed as median and quartile range M (P25, P75). Classification data were analyzed using the chi-square test; quantitative data, t-test; and a logistic regression model, causal relationship between the DCPR and independent variables. We evaluated the diagnostic value of IL-6, mMRC, and CAT score in predicting psychosomatic syndrome in AECOPD patients using receiver operating characteristic (ROC) curves and calculated the area under the curve (AUC), Maximum Youden index, optimal threshold, sensitivity, and specificity. In all statistical analyses, a bidirectional 95% effective interval test was used, and a difference of p<0.05 was considered statistically significant.

## 3 Results

### 3.1 Demographic sociology and clinical data of AECOPD patients

Excluding three cases of inability to cooperate in completing psychosomatic interviews and four cases of inability to cooperate in completing lung function, 202 AECOPD patients were enrolled. Of which, 98 (48.5%) exhibited illness behavior; 37 (18.3%), allostatic overload; and 43 (21.3%), two or more psychosomatic syndromes (**Table 1**).

**Table 1:** Demographic sociology and clinical data of AECOPD patients.

Demographic sociology	
Age, (mean (years) ± SD)	75 ± 8.3
Age range (years)	40-94
Sex (M/F) (N, %)	176/26(87.1/12.9)
Marital status N (%)	
Married	181 (89.6%)
Unmarried	3 (1.5%)
Divorce	5 (2.5%)
Bereave	13 (6.4%)
Education status(mean (years) ± SD)	5.5 ± 3.2
Clinical	
Body mass index, (mean (kg/m^2^) ± SD)	22.7 ± 3.9
Smoking use N (%)	
Never	89(44.1%)
Quitted	61(30.2%)
Current	52(25.7%)
Alcohol intake N (%)	
Never	143(70.8%)
Quitted	31(15.3%)
Current	28(13.9%)
Type 2 diabetes N(%)	36(17.8%)
Hypertension N(%)	125(61.9%)
PaCO_2_(mean(mmHg)± SD)	47.8 ± 13.1
PaO_2_(mean(mmHg)± SD)	96.4 ± 32.9
White blood cell (10^9/^L), (mean ± SD)	8.1 ± 3.9
Neutrophil count (10^9/^L), (mean± SD)	6.3 ± 3.7
Neutrophil ratio (%), (mean ± SD)	73.8 ± 11.8
Lymphocyte count (10^9/^L), (mean± SD)	1.1 ± 0.6
Lymphocyte ratio (%), (mean ± SD)	15.7 ± 9.0
Monocyte count (10^9/^L), (mean± SD)	0.7 ± 0.4
Monocyte ratio (%), (mean± SD)	8.6 ± 3.9
IL-6 (mean (pg/ml) ± SD)	20.9 ± 32.8
Albumin (mean (g/l) ± SD)	36.1 ± 5.2
Prealbumin (mean (mg/l) ± SD)	169.2 ± 70.6
FEV_1_%pred, (mean± SD)	41.6 ± 17.9
FEV_1_/FVC (%), (mean± SD)	53.5 ± 11.7
mMRC (mean± SD)	2.7 ± 1
CAT-score (mean± SD)	21.0 ± 8.1
DCPR(+)	144(71.3%)

PaCO_2_, The partial pressure of arterial carbon dioxide; PaO_2_, The partial pressure of arterial oxygen; IL-6, Interleukin 6; FEV_1_, Forced Expiratory Volume in 1s; FEV_1_%pred, The percentage of predicted values of FEV_1_; FVC, Forced Vital Capacity (FVC); mMRC, modified Medical Research Council; DCPR, The Diagnostic Criteria for Psychosomatic Research.

### 3.2 Comparison of demographic, sociological, and clinical data of AECOPD patients between DCPR (+) and DCPR (-) groups

**Figure 2:**
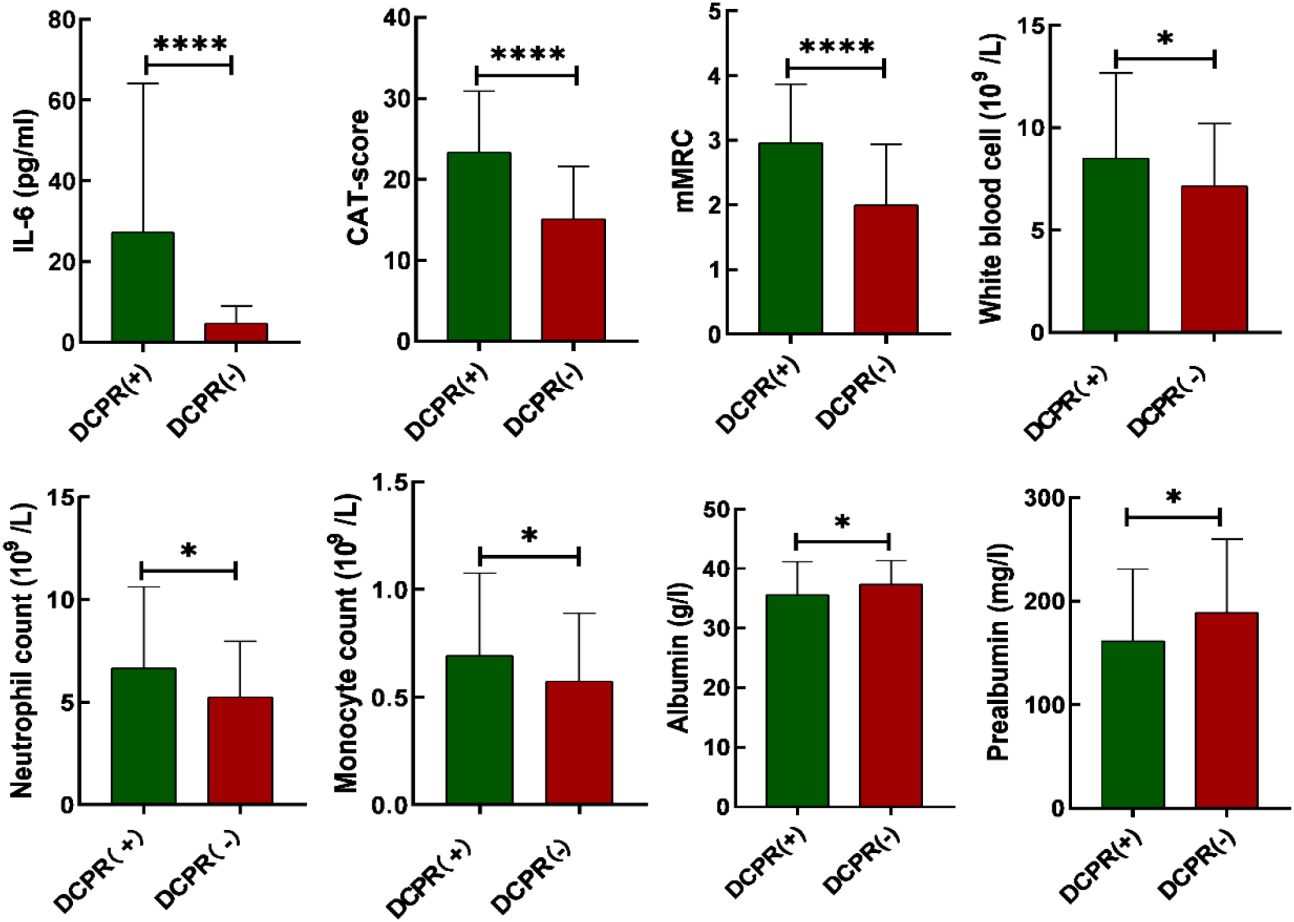
Comparison of bar charts illustrating IL-6, CAT scores, mMRC, white blood cell count, neutrophil count, monocyte count, albumin and prealbumin between patients with DCPR (+) and DCPR (-). DCPR, The Diagnostic Criteria for Psychosomatic Research; IL-6, Interleukin 6; CAT, COPD assessment test; mMRC, modified Medical Research Council; *P < 0.05, ****P < 0.0001.

Among 202 AECOPD patients, 144 were in the DCPR (+) and 58 were in the DCPR (-) groups. The DCPR (+) group exhibited higher white blood cell counts, neutrophil counts, monocyte counts, IL-6, mMRC, and CAT scores than those of the DCPR (-) group, with values of ((8.5 ± 4.2) * 10^9^/L vs. (7.2 ± 3.0) * 10^9^/L, P = 0.026), ((6.7 ± 4.0) * 10^9^/L vs. (5.3 ± 2.7) * 10^9^/L, P = 0.005), ((0.7 ± 0.4) * 10^9^/L vs. (0.6 ± 0.3) * 10^9^/L, P = 0.036), ((27.3 ± 36.8) pg/ml vs. (4.7 ± 4.3) pg/ml, P < 0.0001), ((3 ± 0.9) vs. (2 ± 0.9), P < 0.0001), ((23.3 ± 7.5) vs. (15.1 ± 6.5), P < 0.0001). The DCPR (+) group had significantly lower albumin and prealbumin levels than those in the DCPR (-) group (35.6 ± 6.5). 5.6) g/L vs. (37.4 ± 3.9) g/L, P = 0.026), ( (161.6 ± 69.2) mg/L vs. (188.9 ± 71.0) mg/L, P = 0.014) (P < 0.05). Age, sex, marital status, years of education, BMI, smoking history, drinking history, diabetes, hypertension, PaCO_2_, PaO_2_, neutral ratio, lymphocyte count, monocyte ratio, FEV_1_% pred, and FEV_1_/FVC% were not significantly different between the two groups (P > 0.05; **Table 2**; **Figure 2**).

**Table 2:** Comparison of demographic, sociological, and clinical data of AECOPD patients between DCPR (+) and DCPR (-) groups.

Variable	DCPR 144(+)	DCPR 58(-)	t-test/χ^2^	P -values
Age (years)	75.1 ± 7.9	75.0 ± 9.2	t = -0.08	0.94
Sex (M/F)	129/15	47/11	χ^2^ = 2.7	0.1
Marital status N (Married/Unmarried/Divorce/Bereave)	128/2/4/10	53/1/1/3	χ^2^ = 0.45	0.93
Education status (years)	5.4 ± 3.1	5.6 ± 3.6	t = 0.32	0.75
Body mass index, (kg/m^2^)	22.7 ± 3.9	22.7 ± 3.9	t = 0.1	0.92
Smoking use N (Never/Quitted/Current)	60/48/36	29/13/16	χ^2^ = 2.4	0.3
Alcohol intake N (Never/Quitted/Current)	101/23/20	42/8/8	χ^2^ = 0.16	0.92
Type 2 diabetes N	27	9	χ^2^ = 0.23	0.59
Hypertension N	88	37	χ^2^ = 0.13	0.72
PaCO_2_(mmHg)	45.6 ± 13.8	45.8 ± 11.4	t = -1.46	0.15
PaO_2_(mmHg)	96.4 ± 32.3	96.4 ± 34.6	t = -0.001	1
White blood cell (10^9/^L)	8.5 ± 4.2	7.2 ± 3.0	t = -2.2	0.026
Neutrophil count (10^9/^L)	6.7 ± 4.0	5.3 ± 2.7	t = -2.9	0.005
Neutrophil ratio (%)	74.7 ± 11.7	71.7 ± 12.0	t = -1.6	0.102
Lymphocyte count (10^9/^L)	1.0 ± 0.5	1.1 ± 0.6	t = 1.2	0.25
Lymphocyte ratio (%)	14.8 ± 8.9	18.1 ± 8.8	t = 2.4	0.017
Monocyte count (10^9/^L)	0.7 ± 0.4	0.6 ± 0.3	t = -2.1	0.036
Monocyte ratio (%)	8.7 ± 3.6	8.4 ± 4.4	t = -0.49	0.63
IL-6 (pg/ml)	27.3 ± 36.8	4.7 ± 4.3	t = -7.24	<0.0001
Albumin (g/l)	35.6 ± 5.6	37.4 ± 3.9	t = 2.2	0.026
Prealbumin (mg/l)	161.6 ± 69.2	188.9 ± 71.0	t = 2.5	0.014
FEV_1_/%pred	40.0 ± 17.7	45.5 ± 18.2	t = 2.0	0.051
FEV_1_/FVC (%)	52.5 ± 11.7	55.9 ± 11.6	t = 1.9	0.062
mMRC	3 ± 0.9	2 ± 0.9	t = -6.7	<0.0001
CAT-score	23.3 ± 7.5	15.1 ± 6.5	t = -7.3	<0.0001

PaCO_2_, The partial pressure of arterial carbon dioxide; PaO_2_, The partial pressure of arterial oxygen; IL-6: Interleukin 6; FEV_1_, Forced Expiratory Volume in 1s; FEV_1_%pred, The percentage of predicted values of FEV_1_; FVC, Forced Vital Capacity (FVC); mMRC, modified Medical Research Council; CAT, COPD assessment test.

### 3.3 Multivariate binary logistic regression analysis of risk factors for DCPR in AECOPD patients

The 202 AECOPD patients were divided into DCPR (+) and DCPR (-) groups, with psychosomatic syndrome as the binary dependent variable and white blood cell count, neutrophil count, lymphocyte count, monocyte percentage, IL-6, albumin, prealbumin, mMRC, and CAT scores as independent variables. A multivariate binary logistic regression analysis was conducted, and the results showed that IL-6, mMRC, and CAT score were independent risk factors for psychosomatic syndrome in AECOPD patients, with OR=1.192 (95% CI: 1.091-1.302), P < 0.001), OR=1.922 (95% CI: 1.175-3.144), P = 0.009), OR=1.149 (95% CI: 1.073-1.231), P < 0.001), as shown in **Table 3**.

**Table 3:** Multivariate binary logistic regression analysis of risk factors for DCPR.

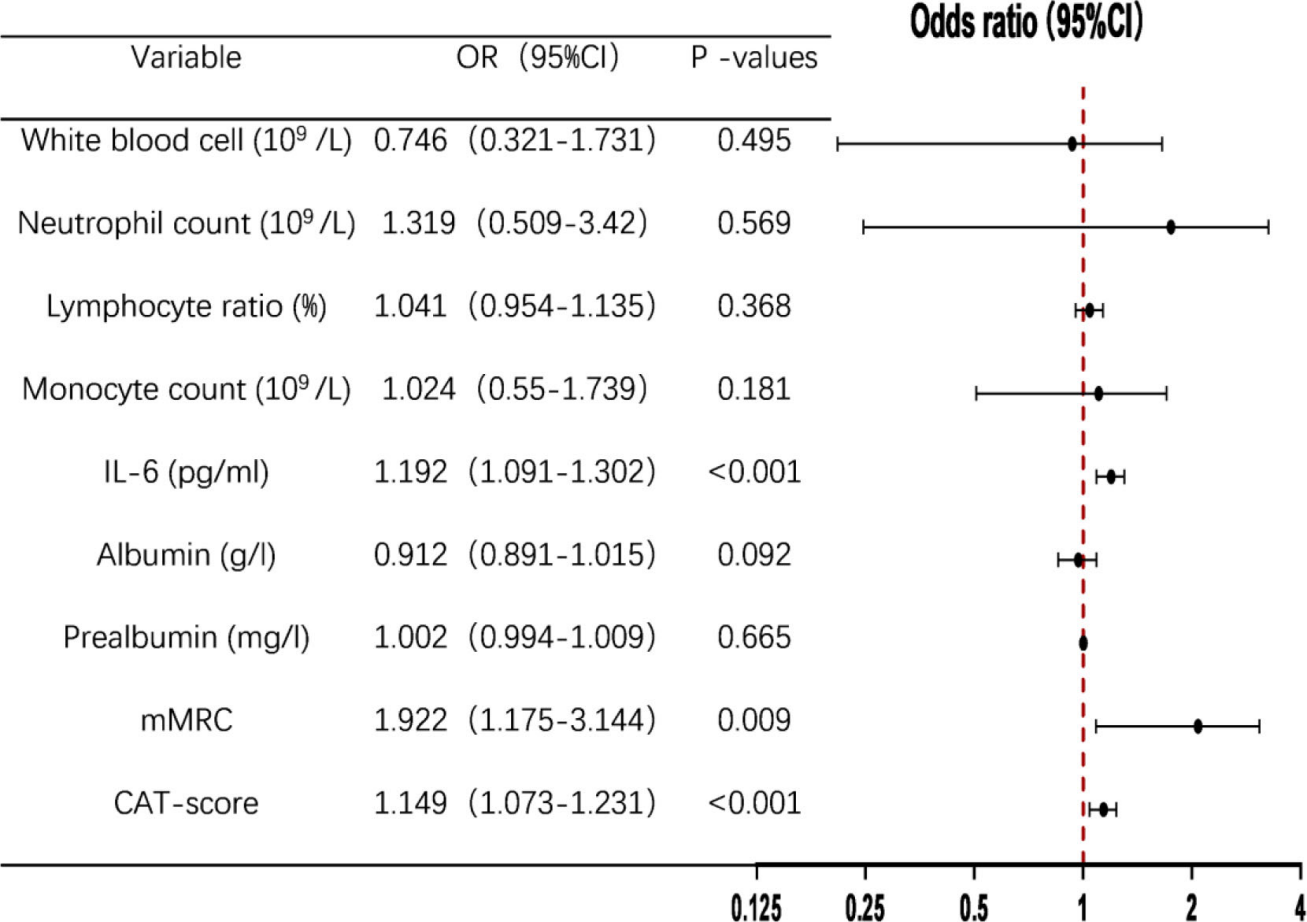

IL-6, Interleukin 6; mMRC, modified Medical Research Council; CAT, COPD assessment test; OR, Odds Ratio; CI, Confidence Interval.

### 3.4 Analysis of predictive factors for psychosomatic syndrome in AECOPD

**Figure 3:**
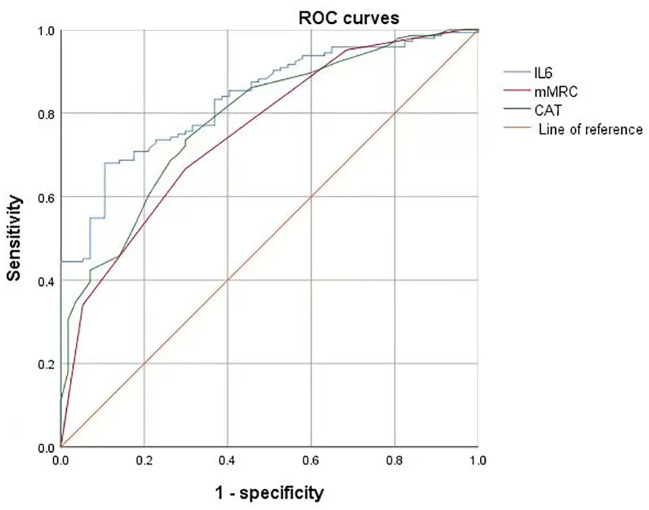
ROC curves for predicting DCPR with IL-6, mMRC, and CAT. ROC, Receiver Operating Characteristic Curve; IL-6, Interleukin 6; mMRC, modified Medical Research Council; CAT, COPD assessment test.

Multivariate binary logistic regression analysis showed that IL-6, mMRC, and CAT scores were predictive factors for psychosomatic syndrome, with good sensitivity and specificity, as shown in **Figure 3**; **Table 4**.

**Table 4:** Analysis of ROC curves for IL-6, mMRC, and CAT scores in predicting DCPR.

Characteristic	AUC(95%CI)	Maximum youden index	Optimal critical value	Sensitivity%	Specific%
IL-6 mMRC CAT-score	0.834(0.778-0.89) 0.755(0.683-0.827) 0.789(0.722-0.856)	0.575 0.374 0.443	7.9 2.5 17.5	68.1 66.7 73.6	89.5 70.7 70.7

ROC, Receiver Operating Characteristic Curve; IL-6, Interleukin 6; mMRC, modified Medical Research Council; CAT, COPD assessment test; AUC, Area Under Curve; CI, Confidence Interval.

## 4 Discussion

Research has confirmed that psychiatric comorbidities are prevalent in AECOPD patients. Among the 202 AECOPD patients enrolled in this study, more than half (144 cases, 71.3%) had psychosomatic syndromes. Specifically, 98 (48.5%) exhibited illness behavior; 37 (18.3%), allostatic overload; and 43 (21.3%), two or more psychosomatic syndromes. The incidence rates can vary depending on the interview skills of clinical doctors and the specific evaluation tools. Through this observational study, we concluded that there is an inherent connection between AECOPD and psychosomatic syndromes, mainly manifested through immune responses triggered by inflammatory factors and symptom burden experienced by these patients. However, to date, the literature on risk factors, such as inflammatory markers and symptom burden, is still limited, which is addressed by our study. We investigated the correlation between AECOPD patients with psychosomatic syndrome and inflammatory factors such as IL-6, white blood cell count, and neutrophil count. We also examined symptoms such as mMRC and CAT scores and their assessed their predictive value for psychosomatic syndrome.

The pathophysiology of COPD and its psychiatric comorbidities are complex. In recent years, many studies have confirmed that inflammatory factors, such as IL-6, white blood cell count, and CRP, play a more significant role in psychiatric comorbidities (34). IL-6 is a pro-inflammatory cytokine that represents the level of inflammatory response in the body. Peripheral pro-inflammatory cytokines enter the central nervous system through humoral, neural, and cellular pathways. Inflammatory factors entering the brain supposedly mainly affect it through the following pathways, leading to psychiatric comorbidities. 1. IL-6 intensifies the stress response by modulating the hypothalamic-pituitary-adrenal (HPA) axis. Specifically, IL-6 directly stimulates the release of corticotropin-releasing hormone (CRH) from neurons located in the paraventricular nucleus (PVN) of the hypothalamus, which in turn facilitates the secretion of adrenocorticotropic hormone (ACTH) from the pituitary gland and cortisol from the adrenal glands (35). Prolonged elevated levels of cortisol subsequently diminish the expression of brain-derived neurotrophic factor (BDNF), resulting in the inhibition and atrophy of the hippocampal nervous system, thereby impairing cognitive and emotional regulatory functions (36, 37). 2. IL-6 Disrupts Tryptophan Metabolism and Neurotransmitter Synthesis, Impacting Emotional Regulation Pathways. IL-6 promotes the expression of indoleamine 2,3-dioxygenase (IDO), which shifts tryptophan metabolism towards the kynurenine (KYN) pathway rather than serotonin (5-HT) synthesis, consequently reducing synaptic 5-HT levels. Furthermore, IL-6 impedes glutamate uptake by astrocytes while activating NMDA receptors, resulting in excitotoxic damage to postsynaptic neurons (38). 3. Central interleukin-6 (IL-6) plays a pivotal role in the activation of microglia, thereby initiating a neuroinflammatory cascade. Upon activation, microglia secrete cytokines, including tumor necrosis factor-alpha (TNF-α) and interleukin-1 beta (IL-1β), which serve to further amplify inflammatory signaling and disrupt synaptic plasticity (39). Concurrently, IL-6 enhances the production of reactive oxygen species (ROS) via the NADPH oxidase pathway, culminating in mitochondrial dysfunction and neuronal apoptosis (40). Evidence from animal models indicates that IL-6 overexpression can inhibit the proliferation of neural stem cells within the dentate gyrus and adversely affect the capacity for emotional recovery (41).

In cases where chronic obstructive pulmonary disease (COPD) is accompanied by hypoxemia, persistent hypoxia upregulates interleukin-6 (IL-6) expression via the hypoxia-inducible factor 1-alpha (HIF-1α) pathway, concurrently exacerbating oxidative stress and mitochondrial impairment within cerebral tissues (42). During acute exacerbations of COPD, there is a marked escalation in pulmonary inflammation, resulting in a rapid surge in IL-6 levels that surpasses the protective threshold of the blood-brain barrier, thereby precipitating central nervous system inflammation (37, 43). IL-6 contributes to the pathogenesis of psychiatric disorders by influencing the hypothalamic-pituitary-adrenal (HPA) axis, altering the metabolism of monoamine neurotransmitters, and promoting neuroinflammatory processes. Current research on psychiatric inflammation suggests that immune system disorders caused by infections promote psychiatric comorbidities (35). Stroll et al. found that elevated plasma IL-6 levels are associated with depressive symptoms in COPD patients and that systemic inflammation may play an important bidirectional role in COPD-related depression (44). Chan et al. confirmed that systemic inflammation leads to an increase in monocytes, neutrophils, and lymphocytes in the blood, thereby upregulating pro-inflammatory processes in immune-related peripheral blood, which contributes to the development of psychiatric comorbidities (45); this was confirmed in the present study. We analyzed the laboratory data of the DCPR (+) and DCPR (-) groups and found that the white blood cell count, neutrophil count, monocyte count, and IL-6 levels in the DCPR (+) group were higher than those in the DCPR (-) group: ((8.5 ± 4.2) * 10^9^/L vs. (7.2 ± 3.0) * 10^9^/L, P = 0.026), ((6.7 ± 4.0) * 10^9^/L vs. (5.3 ± 2.7) * 10^9^/L, P = 0.005), ((0.7 ± 3.0) * 10^9^/L, P = 0.026), (0.4) * 10^9^/L vs. (0.6 ± 0.3) * 10^9^/L, P = 0.036), ((27.3 ± 36.8) pg/ml vs. (4.7 ± 4.3) pg/ml, P = 0.005), and the difference was statistically significant (<0.05). The percentage of lymphocytes in the DCPR (+) group was lower than that in the DCPR (-) group: ((14.8 ± 8.9) % vs. (18.1 ± 8.8) %, P = 0.017), and the difference was statistically significant (P < 0.05; **Table 2**, **Figure 2**). After adjusting for confounding factors such as sex, age, comorbidities, smoking, and alcohol consumption, IL-6 was ultimately identified as an independent risk factor for psychosomatic syndrome (OR 1.192, 95% CI 1.091-1.302, P < 0.001) and an important predictor (AUC = 0.834; 95% CI 0.778-0.89), with a sensitivity of 68.1% and a specificity of 89.5% (**Tables 3** and **4**, and **Figure 3**). However, actually, comorbidities such as type 2 diabetes (T2DM) and hypertension may worsen psychosomatic syndromes through IL-6 and other inflammatory markers (46, 47). This influence will be further studied in the future research.

Inflammation not only plays a crucial role in the comorbidity of psychosomatic syndrome in COPD but also contributes to decreased quality of life also and increased symptom burden, thereby promoting the occurrence of psychiatric comorbidities (48). The symptoms of COPD are primarily characterized by breathing difficulties, and the clinical evaluation of the severity of these difficulties in patients is conducted using the mMRC scale (32). Quality of life mainly includes coughing and sputum production, ability to perform household chores, confidence in going out, sleep, and energy status. CAT is used to clinically evaluate the degree of impact on quality of life (33). A study by Wu et al. confirmed that psychiatric comorbidities in COPD patients are closely related to their subjective symptoms, such as difficulty in breathing, coughing, phlegm, and sleep, and there is no significant correlation with objective indicators such as FEV_1_ (48). Poor subjective emotional feelings of patients lead to a higher incidence of psychiatric comorbidities in COPD (6). Long (49) et al. found that the CAT score was an independent risk factor and a significant predictive factor for AECOPD patients with mental illness (AUC=0.790, 95% CI 0.740-0.834). Increasing evidence shows that the burden of symptoms (including cough, expectoration, wheezing, and chest tightness) has a significant adverse impact on health status, quality of life, and daily activities, leading to an increase in the incidence rate of psychosomatic syndrome and a worse prognosis of the disease (50). Our study confirmed that the mMRC and CAT scores of the DCPR (+) group were higher than those of the DCPR (-) group ((3 ± 0.9) vs. (2 ± 0.9), P < 0.001), ((23.3 ± 7.5) vs. (15.1 ± 6.5), P < 0.001), and the differences were statistically significant (P < 0.05), while there was no statistically significant difference in FEV_1_% pred between the two groups (**Table 2**, **Figure 2**). Multivariate binary logistic regression showed that the mMRC and CAT scores were independent risk factors for psychosomatic syndrome ((OR 1.922, 95% CI 1.175-3.144), (OR 1.149, 95% CI 1.073-1.231)) and important predictive factors ((AUC=0.755, 95% CI 0.683-0.827) (AUC=0.789, 95% CI 0.722-0.856)), respectively (**Tables 3** and **4**, **Figure 3**). This finding is consistent with those of the previous studies.

The levels of albumin and prealbumin in the DCPR (+) group were lower than those in the DCPR (-) group: (35.6 ± 5.6) g/L vs. (37.4 ± 3.9) g/L, P = 0.026), ((161.6 ± 69.2) mg/L vs. (188.9 ± 71.0) mg/L, P = 0.014), with statistically significant difference (**Table 2**; P < 0.05). White matter is an indirect indicator of inflammation, and its synthesis in the liver is inhibited in presence of systemic inflammation (51). Albumin and prealbumin are not used to evaluate nutritional status but rather represent the level of inflammation in the body. Therefore, a decrease in albumin and prealbumin levels indicates occurrence and development of psychosomatic syndromes.

The present study had several limitations. 1.The sample utilized in this study was derived from a single center at Jiading Central Hospital in Shanghai. To enhance the generalizability of the findings, future research should incorporate multi-center and multi-population studies. 2.This investigation employs a cross-sectional observational design, which allows for the identification of correlations between IL-6 levels, symptom burden (as measured by CAT/mMRC), and psychosomatic syndrome, but does not permit the establishment of causal relationships. Therefore, further validation through longitudinal cohort studies or intervention trials is warranted in future research endeavors.

## 5 Conclusions

Our study indicates that elevated levels of peripheral blood leukocytes, neutrophils, lymphocytes, and IL-6, along with decreased monocytes, in AECOPD patients are associated with the development of psychiatric comorbidities. Moreover, subjective symptoms such as cough, phlegm, sleep disturbances, confidence in going out, and difficulty breathing in AECOPD patients contribute more to psychiatric comorbidities than objective indicators such as FEV_1_.

## Data Availability

The raw data supporting the conclusions of this article will be made available by the authors, without undue reservation.
